# Novel Microwave-Assisted Synthesis of COFs: 2020–2022

**DOI:** 10.3390/molecules28073112

**Published:** 2023-03-30

**Authors:** Cristina Rodríguez-Carríllo, Miriam Benítez, Jamal El Haskouri, Pedro Amorós, Jose V. Ros-Lis

**Affiliations:** 1 REDOLI Research Group, Instituto Interuniversitario de Investigación de Reconocimiento Moleculary Desarrollo Tecnológico (IDM), Universitat Politècnica de València, Universitat de València, Doctor Moliner 50, 46100 Valencia, Spain; 2 Institut de Ciència dels Materials (ICMUV), Universitat de València, 46071 Valencia, Spain

**Keywords:** covalent organic framework, microwave-assisted synthesis, COF, 2D, 3D

## Abstract

Covalent organic frameworks (COFs) have emerged as a new type of crystalline porous polymers of great interest. However, their preparation requires long reaction times. Microwave-assisted synthesis (MAS) offers an interesting approach to increasing the reaction rate of chemical processes. Thus, microwaves can be a key tool for the fast and scalable synthesis of COFs. Since our previous review on the topic, the preparation of COFs with microwaves has been evolving. Herein, we present a compilation of COFs studies and experiments published in the last three years on the synthesis of COFs using microwave-assisted synthesis as a source of energy. The articles include imine, triazine, and other 2D COFs synthesized using MAS. The 3D COFs have also been compiled. The chemical structure of the monomers and the COFs and their main parameters of synthesis and application are summarized for each article.

## 1. Introduction

Covalent Organic Frameworks (COFs) have emerged as novel types of materials in recent years. Interest in them grows every year, and more than 1000 articles have been published per year in the last three years (Web of Knowledge). COFs were first described by Yaghi and co-workers in 2005 [1]. They are characterized by the presence of covalent bonds, a crystalline structure, the presence of light elements, and the wide possibilities and versatility of organic chemistry. These characteristics offer high stability, a narrow porosity distribution that can be tailored depending on the monomers, and a high surface area. By varying the nature of the knots, 2D or 3D materials can also be obtained. Because of that feature, COFs have gained increased attention, and great advances have been achieved over the past decade.

Several applications for COFs have been explored, including molecular separations [2], removal of toxic compounds [3], gas capture [4], enzyme immobilization [5], optoelectronic applications [6], analytical chemistry [7], photocatalysis [8,9], electrocatalysis [10], electrochemical sensors [11], batteries [12], and solar fuel production [13].

Various synthesis strategies for the preparation of COFs have been reported. Solvothermal synthesis is widely used. In fact, the first two COFs synthesized by Yaghi and co-workers were prepared by this technique [1]. This methodology allows the combination of high temperature and pressure suitable for the preparation of porous thermodynamic materials. It has been applied classically for the preparation of zeolites. However, solvothermal synthesis can take several days at continuous heating until the reaction is finished, and the selection of the correct solvent is also crucial to success as it governs the solubility of the reagents [14,15]. Ionothermal methodology is an alternative based on the substitution of organic solvent by a molten salt, generally at high temperatures (about 400 °C). It is usually used to generate covalent triazine frameworks using ZnCl_2_ as a solvent and catalyst [16]. Unfortunately, high temperatures and long reaction times increase the risk of decomposition of the reagents and products. Mechanochemistry has emerged as an alternative to prepare COFs at low temperatures. In this case, the monomers are placed in a solvent-free mortar and manually ground at room temperature [14,17]. However, until now, the mechanochemical method has not been very successful in the synthesis of COFs based on Schiff reactions [18]. Other methodologies include multicomponent reactions [19], “two-in-one” synthetic strategy [20], and microfluidic synthesis [21]. To overcome some of these drawbacks, the use of microwaves has attracted wide interest in recent years due to their advantages of shorter reaction times (reaching 200 times faster values) and higher reproducibility [22], being mainly applied to the transmission of information and for heating. They have been widely used in organic and inorganic synthesis applied to nanomaterials [23,24], including metalorganic frameworks (MOFs) [25], zeolites [26], silicas [27], mesoporous materials [27], and others.

Over the years, the number of reviews on COFs has increased significantly. The relevance of the topic prompted us to prepare a comprehensive review of the use of microwaves for the synthesis of COFs [28]. Since then, no other review has centered on the topic despite the evolution of the discipline. We present here an update of the review of the publications in the period 2020–2022, with a discussion about the synthesis procedures, main applications, and future perspectives. We expect that this review will improve the use of microwave-assisted synthesis for the preparation of COFs.

## 2. Structure and Characteristics of COFs

As mentioned above, COFs are a class of crystalline porous polymers. Compared to other crystalline porous materials such as Metal Organic Frameworks (MOFs), these include exclusively organic subunits of light elements (H, B, C, N, O, Si) connected by strong covalent bonds, forming 2D or 3D materials. One of the advantages of these materials is that the structural composition, topology, porosity, and chemical reactivity can be predicted considering the units involved. We detail below a list of the most remarkable monomers, linkages, and structures of COFs.

### 2.1. Monomers and Linkages

**Figure 1:**
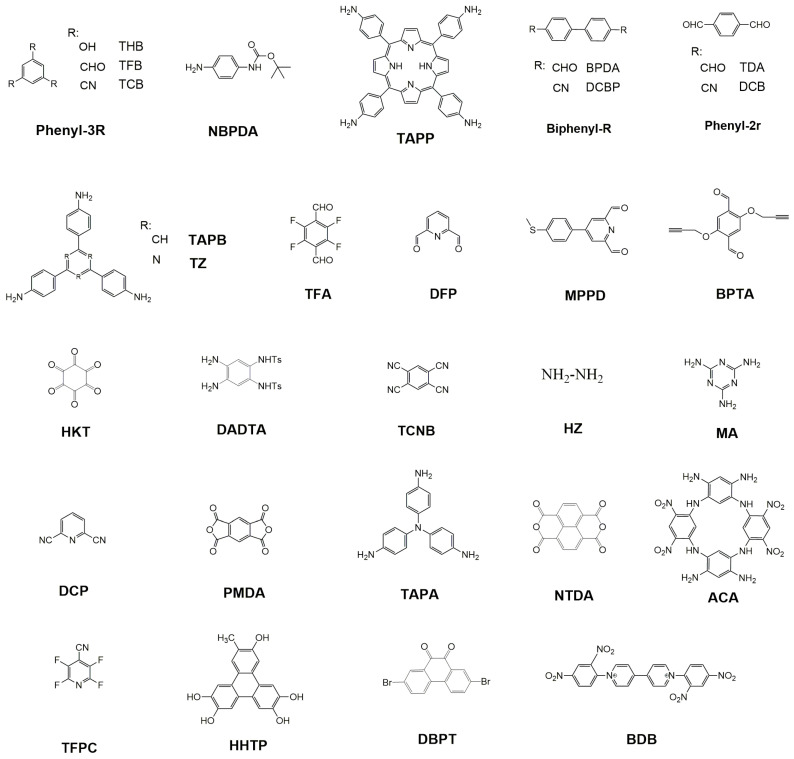
Monomers used in the recent papers discussed in this review.

The monomers are diverse, in agreement with the variety of COFs reported in the literature. Figure 1 contains the monomers used in the materials included in this review. As can be seen, they show certain similarities. The first kind of monomers with a trigonal or tetrahedral symmetry acts as the vertex of 2D and 3D COFs, respectively. They are complemented in several cases with linear monomers that are sited between the vertex molecules, increasing the pore size. Both kinds of monomers are rigid molecules to ensure the crystallinity and rigidity of the final material and, in the case of 2D COFs, tend to include aromatic plane molecules to facilitate layer-stacking.

The functional groups commonly found in COFs include aldehydes, cyanides, amines, catechol, pyridines, thiols, amides, alcohols, and succinic anhydride moieties. The groups react through condensation reactions in which a small molecular byproduct (e.g., water) is generated. The linkages presented in this review include imines, triazines, and succinimides. Other common bonding groups are B–O (boroxine, boronate ester), B=N (borazine), C–N (β-ketoenamine, imide, imine, and amide), N=N (azodioxy), and B–O–Si (borosilicate). The chemical nature of the linkage is a key issue because it strongly influences the stability and chemical resistance of the COF. For example, the first boron-containing COFs described are very sensitive to humidity. In contrast, the imide of triazine-based materials is more resistant. 

### 2.2. Topology

**Figure 2:**
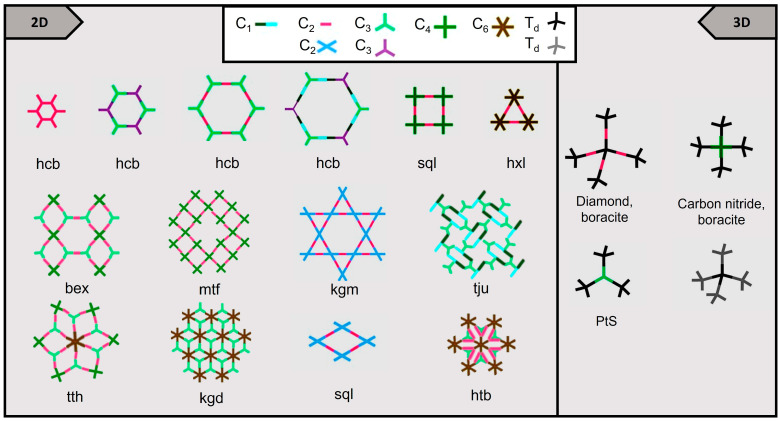
Topologies of COFs.

A unique feature of COF compared to other porous solids is that its framework can be designed predictably at three different structural levels: pore, framework, and both pore and framework complementary design [29]. The formation of 2D or 3D COFs is determined by the position in which the growth of the forming blocks occurs (Figure 2); additionally, the size of these units will influence the pore size. A rigid structure and a specific geometry are the sole conditions that the subunits must meet [29].

There are many topologies for COFs, but they all can be classified into a few general groups [30,31]. Two-dimensional COFs have different topologies. As shown in Figure 2, their nature depends on the number of monomers and functional groups, but most of them are based on symmetric monomers. Recently, the list has been expanded with the incorporation of the tju topology found in COFs produced by self-condensation of asymmetric K-shaped metal-Salphen monomers [31]. By contrast, 3D COFs are characterized by a tetrahedral center [22].

## 3. General Notions of Microwave-Assisted Synthesis

One of the differential characteristics of COFs in comparison with other organic polymers is the narrow porosity distribution associated with their crystalline character. In general, this crystallinity is achieved after an aging process in which the structural extended order is produced due to the dynamic covalent chemistry (DCC) process [32]. DCC relies on the reversible formation of covalent bonds between molecules. This equilibrium provides COFs a way to remediate kinetically generated defects. In such a way, they can be repaired, allowing the formation of highly ordered frameworks. We can indicate that COFs are thermodynamic products favored at high temperatures and/or long reaction times [18]. These reaction times are usually provided in the solvothermal method for COFs preparation [33]. Microwave synthesis is a suitable alternative to the solvothermal methods because it provides a way to quickly synthesize COFs under solvothermal conditions with similar or improved properties compared to conventional methods [28]. To understand the benefits of microwaves for the synthesis of COFs, it is advisable to have a general vision of the nature of microwaves, their interaction with matter, and their advantages. 

Microwaves (MWs) are a form of electromagnetic radiation lying between infrared and radiofrequency. To date, they have mostly been applied for transmitting information and for heating [34]. Microwave frequencies vary between 300 MHz and 300 GHz (wavelengths from 1 m to 1 mm, respectively), but 915 MHz, 2.45 GHz, and 5.85 GHz are commonly used for Industrial Scientific and Medical (ISM) applications. 

We can find three kinds of microwave interactions with matter: transmission, reflection, and absorption. Transmission is the behavior typical of materials transparent to MW, such as Teflon. Reflection is observed in materials like metals that do not allow MW to pass through them. In both cases, there is no transference of energy from the MW to the matter. The third kind of interaction, absorption, is relevant from a chemical point of view. Electromagnetic energy is absorbed to some extent by the material and converted into heat [35]. The absorption depends on the nature of the material and the frequency. In general, for nonmagnetic materials, the heating of the materials is due mainly to the electric component of the MWs. The complex permittivity ε* is defined as a measure of the ability of a material to absorb and store potential energy. It has two components: the permittivity ε′, also known as the dielectric constant, which describes the ability of a material to act as a capacitor storing energy; and the other component, the loss factor ε″, which reflects the ability of the material to dissipate energy [35].

Two main mechanisms have been described to explain the MW-induced heating of materials: dipolar polarization and ionic conduction. The former is based on the effect of the electric field to orientate polar molecules. The dipoles tend to align with the electric field, but, if the frequency is too high, they cannot orientate and generate friction between them that is converted into heat. The latter takes place when mobile charge carriers (electrons, ions, etc.) move through the material under the influence of the microwave electric field. The induced electric current collides with neighboring molecules or atoms, thus creating an electrical resistance that further heats the material [36]. 

Furthermore, some literature has referred to “microwave effects” to describe some controversial phenomena associated with microwave irradiation that cannot be easily explained, attending to differences in temperature between microwave and conventional heating [37]. These non-thermal effects include an enhanced reaction rate, improved product yields and mechanical properties, reduced processing/curing time, reduced activation energy, and different reaction pathways [38]. However, these seem to be derived from an incomplete understanding of the actual electromagnetic theory, in which an accurate mechanism of microwave interactions with matter is still not effectively explained by existing theories [39].

One of the main advantages of MWs as a source of energy in chemical reactions is that the energy is generated directly in the sample by absorption. It avoids the necessity of conventional heating methods like sand, oil baths, electric heating, or heating jackets. Thus, the process is more energy efficient because it is unnecessary to heat the full system. Another advantage is that the MWs penetrate the sample, transferring heat in a much faster way than the other methods in which heat is transferred by convection from the surface and greatly depends on the inner thermal conductivity of the sample. As MWs penetrate the sample, the temperature profile is inverted, and samples are efficiently heated from the inside to the outside. As a result, much more homogeneous temperatures are achieved in shorter times when MWs are applied, resulting in well-controlled reaction conditions. In this way, MWs produce better temperature profiles than conventional heating techniques. [40]. 

Thus, the remarkable advantages of MWs in chemistry include higher synthesis rates and shorter reaction times (from hours to minutes), homogeneous products, better properties, higher yields, lower power consumption, and others. Due to these advantages, it is not surprising that MWs have been used in the synthesis of COFs [27].

## 4. Microwave-Assisted Synthesis of COFs

Depending on their structural and geometrical aspects, COFs can be found in two-dimensional (2D) or three-dimensional (3D) frameworks [41]. Two-dimensional COFs, formed by building blocks linked by covalent bonds producing polymeric layers, are the most common, with more than 200 2D-COFs reported [42]. Herein, we will discuss the most remarkable features and applications of COFs produced under MAS (microwave-assisted synthesis). For this purpose, a classification has been made depending on the type of bond formed between monomers and their 2D or 3D nature. A summary of the analysis and the synthesis conditions is presented in Table 1.

### 4.1. Imines- and Enamines-Based 2D COFs

**Figure 3:**
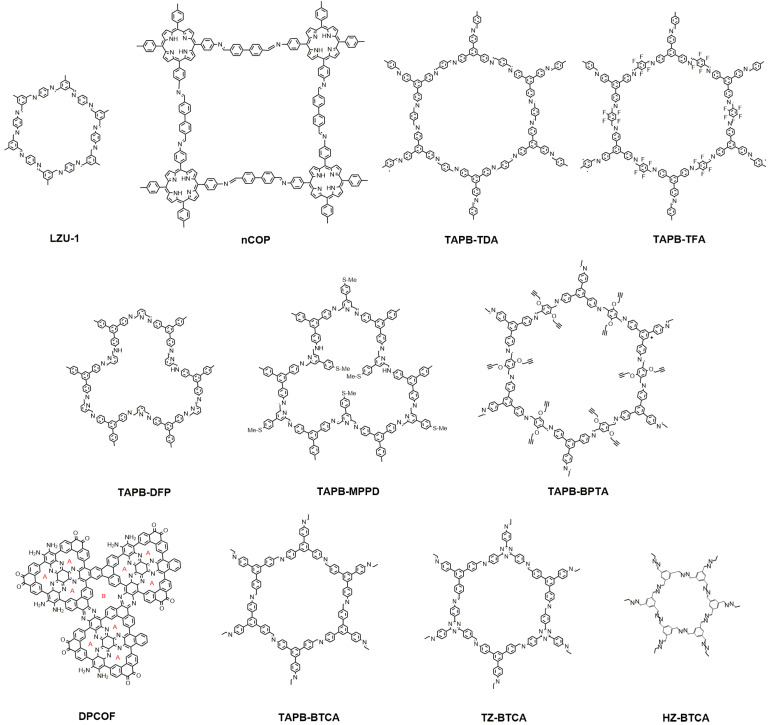
Structure of the imine- and enamine-containing COFs.

Imine-linked COFs are a series of materials widely explored due to their easy preparation, versatility, and chemical stability (in particular, in relation to water and weak acid/base solutions) that make them especially suitable materials for the development of gas adsorbents, sensors, catalysts, and water purification [59,60]. Typically, aryl amines and aldehydes are condensed under solvothermal conditions using Brønsted acids as the catalysts to produce imine-linked COFs [61]. Synthesis temperatures around 120 °C are typical solvothermal conditions and last for several days in a sealed tube until the condensation reaction is complete. Researchers have made numerous attempts to find quicker, more repeatable, and easier synthetic routes to prepare nitrogen-linked COFs because they represent a very promising molecular source of 2D materials [62], avoiding the high temperatures and long synthesis times used on the conventional solvothermal routes. The COFs reported in the period 2020–2022 containing imines or enamines are described in Figure 3.

In 2017, 1,3,5-Triformylbenzene (TFB), 4-(tert-butoxycarbonylamino)-aniline (NBPDA), and poly(N-vinylpyrrolidone) (PVP) were dissolved in ethanol and trifluoroacetic acid to create COF LZU-1 with hexagonal structure by the microwave-assisted synthesis in only 30 min at a temperature of 120 °C [63]. Guntern et al. developed this procedure to prepare colloidal COF/Nanocrystal Hybrids (NC@LZU1) [43]. The particle size was regulated with the addition of PVP as a passivator of the surface and reaction time obtaining particles from 100 to 600 nm. Since LZU1 grows over the NC, the inner layers show a more amorphous character, and the material enhances the crystallinity with the reaction time and particle size increase. Although most of the materials reported are based on Au nanoplates as NC, the authors report that this approach can be applied also to other nanoparticles, such as WO_3_ nanowires and Fe_3_O_4_ nanospheres. Furthermore, the process can be repeated with subsequent loading of nanocrystals and growth of the COF material to synthesize NC@LZU1@NC@LZU1 core− shell−core−shell structures in the form of colloidal solutions. The same procedure (NBPDA and TFB in ethanol, PVP, and TFA heated under microwave irradiation to at 120 °C for 30 min) was used to prepare the LZU COF that was modified in a second step to prepare an LZU COF functionalized with PEI or cysteine moieties (LZU-PEI and LZU-Cys respectively). In this case, the synthesis took advantage of the aldehyde groups present in the reaction mixture able to condensate with the amino groups of PEI or cystamine. The resulting materials have a layer of positively charged amines that reduce the particle size (aggregation) and increase the Z potential, improving the suspensibility of the COF by electrostatic repulsion between particles. The materials show low toxicity at the cellular level, an excellent gene transfection ability confirming the permeation of the cellular membrane, and the potential to be applied as an antitumoral agent [44]. 

Another example of COF for biomedical applications prepared by MAS is nCOP. This material is synthesized by Schiff base condensation of meso-tetrakis(4-aminophenyl), porphyrin (TAPP), and 4,4′-Biphenyldicarboxaldehyde (BPDA) with microwave heating for 20 min, using PVP as a capping agent. The material was loaded with the bioactive molecule 1-methylpropyl 2-imidazolyl disulfide (PX-12), a thioredoxin-1 inhibitor able to induce apoptosis in tumor cells under photodynamic therapy simply by contact in solution. This process is spontaneous, probably due to the hydrophobic or aromatic interaction between the COF and the guest molecule. One of the advantages of this kind of COF is that it presents absorption and fluorescence (680 nm) in the visible zone. Thus, it can be followed easily with common spectroscopic techniques. The nCOP-PX-12 was used for acid pH-controlled release common in the hypoxic environment of tumors, due to the partial hydrolysis of the imines and shrinkage of the nanoparticles at pH 5 that releases PX-12 and TAPP hydrolyzed from nCOP. The nanosystem exhibits excellent tumor accumulation and biosafety, with up to 98.5% of cancers inhibited [45].

Another microwave-assisted method for the preparation of imine-COFs is described for the hexagonal materials TAPB-TDA-COF and TAPB-TFA-COF [46]. These materials combine the aromatic triamine tris-aminophenyl benzene TAPB and the aromatic aldehyde TDA (TAPB-TDA-COF) or its fluorinated derivative TFA (TAPB-TFA-COF). In this case, the synthesis process is longer and more complex. The irradiation time is 1 h in a closed vessel and includes a mixture of solvents and acetic acid as a catalyzer, the previous ultrasonication of the reagents, several washing steps post-synthesis, and drying overnight. The potency is relatively low (200 W), but the material is obtained in a good yield (>85%). Unfortunately, the surface area is lower than 200 m^2^ g^−1^, indicating that the crystallinity of the material can be relatively low. The authors assign this behavior to the short reaction time; in fact, the area doubled when the reaction time doubled. The authors were interested in applying these materials as hydrophobic materials for oil/water separation. The F groups introduced into the benzene ring promoted hydrophobicity and stability of the COF. The TAPB-TFA-COF powder exhibited excellent water-in-oil emulsions separation performance and can be used in several cycles. This result suggests a use to treat oil spills and the separation of industrial water-in-oil emulsions. 

Das et al. have developed a microwave-assisted synthesis to prepare another COF able to capture organic solvents [47]. It also uses TAPB as a knot, in which case the aldehyde liker includes a pyridine ring as an aromatic unit. Apart from the material with 2,6-diformylpyridine (DFP) (TAPB-DFP), another material including 4-(4-(methylthio)phenyl) pyridine-2,6-dicarbaldehyde (MPPD) has been used to include a thianisole functional group that will confer hydrophobicity to the COF structure (TAPB-MPPD). The presence of phenyl groups, as opposed to alkyl chains, provides additional π-stacking between layers and, thus, better crystallinity. The COFs were obtained in a reaction time of 2 h under microwave irradiation with a yield close to 90%. Both materials are thermally stable up to 400 °C; however, as expected, the functionalization significantly reduces the pore volume (0.55 and 0.08 cm^3^ g^−1^ for TAB-DFP and TAB-MPPD, respectively). TAPB-DFP is hydrophilic; by contrast, the presence of the methylthiophenyl group increases the hydrophobicity of the material. TAB-MPPD can capture (>500 wt%) for various types of oils, and the absorption is reversible (beyond 10 cycles).

The same authors have gone one step further in the use of COFs for separation and purification purposes by their incorporation into a membrane [48]. In this case, the COF employed is TAPB-BPTA, similar to the previous ones, but including two alkyne groups in the dialdehyde to facilitate the functionalization. The synthesis procedure was like that reported for TAPB-TFA-COF above (200 W 1 h of irradiation). The material was grafted with cysteine by a radical click reaction to obtain a COF with aminocarboxylate groups (TAPB-BPTA-CYS). The crystallinity of TAPB-BPTA and TAPB-BPTA-CYS was confirmed by powder X-ray diffraction analysis. The carboxyl and amino groups in TAPB-BPTA-CYS improve the dispersibility in the polyethyleneimine coating solution and react with the acyl groups in trimesoyl chloride added as a copolymer. The membrane was prepared by a coating of a PSf ultrafiltration membrane. The COF coating enhances the separation properties, and this capability increases with the size of the molecules.

DPCOF has been included in this section because the material is prepared to depart from aldehyde and primary amines [49]. This is a novel material that contains a dual porosity system that can be used to coordinate transition metals (A in the DPCOF structure of Figure 3), such as iridium or platinum, and to capture small gas molecules (B in the DPCOF structure of Figure 3) simultaneously. The product is prepared in three steps, two of them including microwaves. In the first step, hexaketocyclohexane (HKT) condensates with 1,2-diamino-4,5-ditosylamido-benzene (DADTA), incorporating tosyl-protected amines. The resulting product is then coupled with dibromo-9,10-phenanthrenequinone (DBPT) to form the trimer with the metal coordinating cavities that are the monomer for the 2D-COF formation. When the monomer is exposed to an acid medium in the presence of microwaves, the amino groups are detosylated and react with the quinones, obtaining the dual pore DPCOF. Alternatively, the material can incorporate Ir(III), Pt(II), and Ni(II) if the monomer is exposed to the corresponding salt under microwave heating at 145 °C for 1 h previously to the formation of the COF. Compared to other 2D materials where metals are incorporated using a post-synthetic strategy, DPCOF allows the introduction of the functional metals directly into the monomer before COF formation. The result is a fully metallated COF that is decorated in a completely ordered array that opens the door towards a new family of materials by variation of the transition metals. 

Finally, imine COFs can also be prepared following green chemistry principles [50]. Three COFs were prepared: TAPB-BTCA, TZ-BTCA, and HZ-BTCA in one pot with good yields, avoiding organic solvents under mild conditions. The authors compare the materials produced with and without microwave irradiation. MAS reduces the reaction time from days to 5 h, maintaining the crystallinity, with only a slight reduction in the surface area.

In conclusion, this section lists the reported techniques for producing C-N-based COFs using microwave-assisted synthesis. Also discussed are methods to enhance crystallinity during COF production in microwave settings. Microwaves create materials of equal or superior quality, have a high BET surface area, and are resistant to aqueous conditions. They also considerably shorten the time needed for synthesis, from days to hours.

### 4.2. Triazine-Based 2D COFs

**Figure 4:**
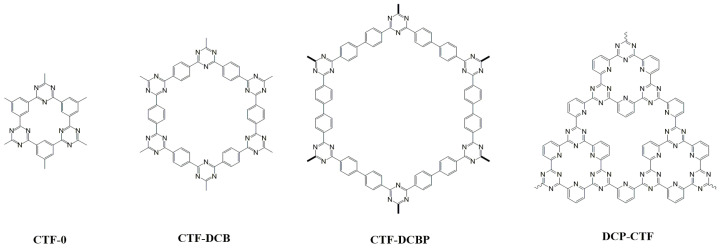
Structure of triazine-containing COFs reviewed.

Covalent triazine-based frameworks (CTFs) show outstanding properties related to a high N content, planarity, and electron delocalization. For example, their properties as semiconductors with adjustable band gaps have been recently applied for photocatalytic water splitting. They were first reported in 2008 by Kuhn et al. in an ionothermal synthesis in which nitriles were trimerized using ZnCl_2_ as both solvent and catalyst [64]. However, high temperatures (400–700 °C) and long reaction times (40 h) were required, producing decomposition, to some extent. These materials can also be prepared using previously formed triazines in the reagents that are incorporated into the final CTF structure by the reaction of other functional groups, forming linkages such as imine and imide. The first method, consisting of the direct formation of triazine rings from nitrile groups, is the most commonly employed in the microwave-assisted synthesis of triazines. Figure 4 shows the structure of the triazine materials prepared in recent years.

Ren et al. developed a synthetic method in which ZnCl_2_ was substituted by trifluoromethanesulfonic acid (TFMS) as a molecule with high catalytic activity and strong polarity to capture and transfer the MW energy to the nitrile-based monomers to greatly facilitate the continuous cyclotrimerization reaction [65]. Using TFMS, Zn-free CTFs could be produced in shorter times at much lower reaction temperatures. A similar procedure was used for the preparation of CTF-0 using 1,3,5-tricyanobenzene (TCB) with diverse quantities of TFMS under microwave irradiation for 30 min at 110 °C followed by washing and drying in a vacuum overnight at 180 °C [51]. The optimum ratio was obtained for a 1:3.5 TCB:TFMS molar ratio. The CTF-0 synthesized by MAS shows a high photocatalytic activity under both UV and visible light for water-splitting. Hydrogen generation is higher for the material prepared under microwave irradiation (7 mmol H_2_ h^−1^ g^−1^). By contrast, the material prepared by the ionothermal method is most efficient for oxygen evolution. These results indicate that the band positions and the interlayer stacking structures of CTF-0 were modulated by varying synthesis conditions. In addition, MAS can be more adequate for H_2_ generation because it produces the most ordered interlayer structure and the highest amount of triazine units. 

Going a step further with a similar synthesis approach, a conventional household microwave was used, and further information about the reaction and its scalation was obtained. CTF-0, CTF-DCB, and CTF-DCBP were prepared using TFMS [52]. CTF-DCBP was prepared for the first time. Again, the molar ratio of TFMS to monomers, reaction time, and microwave power were found to have a great influence on the crystallinity, color, and specific surface area of obtained products. The powder X-ray diffraction (PXRD) patterns of the three triazine COFs displayed very narrow and intense peaks that agree with the simulated values confirming the crystallinity and structure. The most remarkable features of this synthesis procedure are the short reaction time (20 min) and the possibility to scale the batch to obtain more than 100 g. The authors also studied the polymerization and crystallization of the triazine COFs, finding that the monomers rapidly polymerize into periodic 2D molecular sheets in 10 s during the nucleation process and then further grow into more ordered framework structures over the short microwave course. Also, the materials are exfoliated simply by ball-milling to crystalline single-layer/few-layer 2D triazine polymer nanosheets, improving the hydrogen evolution rate. 

MAS can be used for the post-synthetic modification of triazine COFs. In fact, microwave-assisted thermal decomposition reactions of metal complexes are established methods for the synthesis of metal NPs. Palladium nanoparticles and iridium oxides have been prepared on 2,6-dicyanopyridine-based CTF (DCP-CTF) [53]. Either Pd(acac)_2_ or Ir_4_(CO)_12_ were dispersed with DCP-CTF and were irradiated at 100 W for 20 min (Pd-NP) or 30 min (IrOx-NP). The metal loading was homogeneous within the COF particles and was higher in Pd samples (29 wt%) than in Ir (14%), indicating unreacted metal particles in the dispersion. Average particle sizes around 13 and 2 nm were determined for Pd and Ir-containing samples, respectively. The CTFs act as molecular stabilizers for nanoparticles, improving their electrocatalytic properties toward hydrogen evolution reaction (HER) and the oxygen reduction reaction (ORR), compared to commercial platinum catalyzers.

### 4.3. Other 2D COFs

**Figure 5:**
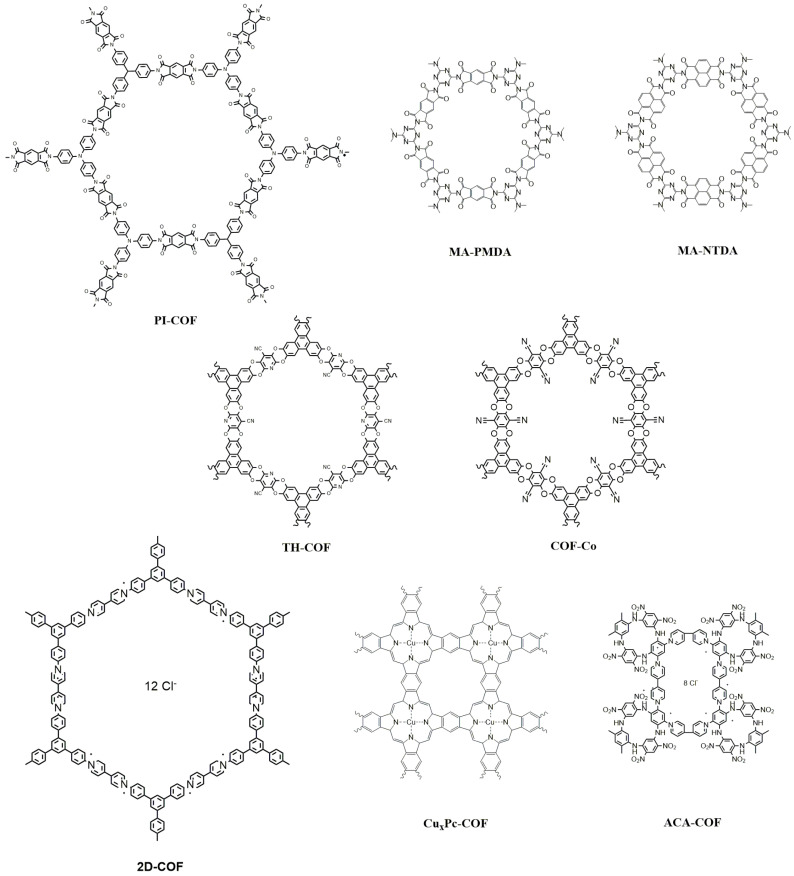
Structure of other COFs reviewed.

In addition to the most common functional groups mentioned above, other groups such as esters, imides, amides, or direct C—C coupling have been used in the microwave-assisted synthesis of bidimensional COFs. Although they occur in the bibliography much less frequently than those bonds collected in previous sections, these linkages are very useful in the way they offer the opportunity to synthesize a wide variety of materials with different applications and with properties even superior to those discussed so far. A summary of the structure of the COFs discussed in this section can be found in Figure 5. 

Imides are an N-containing functional group formed by the reaction of an amine with an anhydride. Lee et al. prepared polyimides with different compositions under a microwave irradiation method [66]. In particular, the authors reacted pyromellitic dianhydride (PMDA) with tris(4-aminophenyl)amine (TAPA) to produce a highly crystalline COF (PI-COF) with remarkable thermal stability. The reaction time was greatly reduced due to microwave irradiation, from five days, necessary under conventional solvothermal conditions at 200 °C, to just 2 h at the same temperature under MAS, resulting in solids with comparable physicochemical parameters. One recent study focuses on the pitfalls associated with the synthesis of polyimide-linked two-dimensional covalent organic frameworks [54]. It evaluates the effect of variations in reaction temperature, time, heating method, and monomer structures to establish whether the formation of ordered crystalline material is occurring. With the use of microwaves, melamine (MA) and PMDA react in N-methyl pyrrolidone (NMP) as solvent at 200 °C in only two hours. This result implies a significant time shortening compared to the three days required in the conventional method. The heating method influences the shape of the particle: a thick 3D rhombohedral structure across multiple layers for the conventional method vs. well-defined thin flakes that exhibit electron diffraction for the MAS. FT-IR indicates that MAS displayed stronger carbonyl stretches, as expected for the phthalimide functionality of the MA-PMDA COF. In addition, noticeable differences were observed between thermal and MW conditions in the formation of the PI-PMDA. By contrast, in the preparation of the MA-NTDA COF, a highly ordered material, appeared for both heating systems, though the type of organic materials strongly influences it. XRD indicates that using salts as precursors does not allow the formation of COF.

Metal phthalocyanines can also generate 2D COFs [67]. Liu et al. explored the synthesis and application of copper phthalocyanine-based covalent organic frameworks (CuPx-COF) for highly efficient radioactive iodine capture. The material is prepared in only 15 min with a MAS procedure departing from 1,2,4,5-tetracarbonitrilebenzene (TCNB) as the only organic ligand and a copper salt. Strong XRD peaks and FT-IR confirm the crystallinity of the material and the COF structure. However, the surface areas below 50 m^2^ g^−1^ are indicative of the low porosity due to the blockage of the pores by the metal cations. The iodine capture is driven by the charge transfer arising from nitrogen-rich phthalocyanine structures and electron-rich π-conjugated systems with iodine molecules. Moreover, the strong electrostatic interaction between Cu(II) on chelate centers and polyiodide anions also plays an important role in firmly trapping radioactive iodine.

Dioxin-linked COFs are another family of stable materials due to the strong nature of their covalent bonds. However, synthesizing COFs via irreversible reactions (i.e., nucleophilic substitution) is more challenging than synthesizing reversible reactions. For example, TH-COF was synthesized via MAS by a combination of 2,3,5,6-tetrafluoro-4-pyridinecarbonitrile (TFPC) and 2,3,6,7, 10,11-hexahydroxy triphenylene (HHTP) under microwave heating at 100 W at 70 °C for 30 min [55]. The material has an extraordinarily large surface area of 1254 m^2^ g^−1^. The FT-IR spectra of TH-COF present the characteristic dioxin and C≡N bands, and XRD confirmed the crystallinity of TH-COF. Notably, this COF resists different organic solvents, 6 M HCl, and temperatures up to 370 °C. TH-COF-coated fibers have demonstrated their use for the solid-phase microextraction (SPME) of perfluorinated alkyl substances (PFASs). These characteristics largely stem from the extraordinarily large surface area of TH-COF yielded via microwave-assisted irreversible synthesis and the exceptional intermolecular hydrogen bonds between the pyridine N atoms of TH-COF with PFASs. Microwaves can also be used for the formation of cobalt complexes with the nitrile groups of TH-COF [56]. Loading with the metal induces a slight reduction in the peak intensity and a shift to a smaller angle in the XRD patterns of COF-Co, which should probably be ascribed to a reduction in the crystallinity and a slightly enlarged distance of adjacent two COF, respectively. The material can be used for energy storage as an anode for high-performance organic-potassium batteries because the presence of Co between the layers of the COF induces defects that enhance the π-K^+^ interactions, creates more exposed active sites, and improves K^+^ insertion/extraction kinetics.

Health applications are becoming an area of interest for COFs as noted in the papers mentioned above. The capture of dialysis waste products is another use. The material combines a bispyridinium salt (BDB) with an azacalix [4] arene (ACA) following the Zincke reaction under irradiation of microwave materials at 90 °C for 3 h to generate the cationic ACA-COF [57]. Another ACA-COF was synthesized in solvothermal conditions, and the crystallinity observed was higher using microwave irradiation. The porosity, composition, and flexibility make the resulting ACA-COF a good absorber of both biomolecules and contaminants in water. ACA-COF retains uric acid and creatinine, two major waste products generated during hemodialysis treatment in patients with renal failure. The positive charges interact with the uric acid and the aromatic ring of the calixarene with the creatinine. The authors highlight that the absorption rate is several orders of magnitude faster than reported values.

The inclusion of bispyridium salts in the structure has also been explored in 2D-COF [58]. The π-conjugated viologen-COF (2D-COF) can be synthesized through the covalent integration of 1,1′-bis(2,4-dinitrophenyl)-[4,4′-bipyridine]-1,1′-diium dichloride (BDB) and an aromatic amine 1,3,5-tris(4-aminophenyl)benzene (TAPB) via the microwave-assisted Zincke reaction for 2 h at 100 °C, using ethanol and water as solvents. One of the most notable achievements of this paper is the preparation of the COF in situ inside a polymer membrane in the absence of any solvent. Microwave radiation enables the formation of uniformly distributed 2D COFs in the interconnected polymer network and impedes the aggregation between COF layers. The size of the COF particles increases with the concentration of precursors (of up to 0.85 μm). The membrane containing the 2D-COF is brown, flexible, and can be curled and folded by hand. The result is a material with significantly improved thermal stability and gas separation performance with a 186% increase in CO_2_ permeability and up to 177% increase in selectivity compared with the plain membranes.

Microwaves can also help to develop new processing methodologies to enable inorganic/organic heterostructures [68]. Electromagnetic radiation is the key element in the selective, scalable, and versatile deposition of few-layer COFs onto monolayer transition metal dichalcogenides. The microwave power influences the optoelectronic properties with increasing hybridization and electronic interactions as microwave powers are increased to above 100 W. 

In conclusion, microwaves have effectively given COFs a wide variety of bonds, reducing time compared to the traditional solvothermal approach. They have also been used with COFs that contain charged moieties and counterions, which have an interesting effect on final structure and characteristics.

### 4.4. 3D COFs

As the examples reviewed have shown, 2D COFs are the most common type. Although 3D COFs possess promising properties derived from the linkages extended in the three dimensions of the space, such as high porosity and low density, the limited choice of tetrahedral building blocks and other related synthetic difficulties have hampered their development [42], and only a relatively low number of examples have been reported to date. Moreover, the number of publications focused on 3D COFs produced under microwave irradiation is drastically lower than those for 2D COFs. COF-102 was the first 3D COF synthesized in a microwave oven in 2009 [69]. In recent years, no advances have been reported in this area. 

## 5. Summary and Expectations

Covalent Organic Frameworks (COFs) are a type of crystalline porous materials that present a mesoporous structure that confers properties and applications like other materials such as mesoporous silicas or metal-organic frameworks (MOFs). In addition, 2D-COFs exhibit properties like other two-dimensional materials. However, in many cases, these materials require time-consuming synthesis procedures, in some cases taking several days. The use of microwaves for chemical synthesis has been a successful strategy exploited for decades to obtain products much more quickly that are more homogeneous and reproducible, sometimes offering better performance and properties (for example, porosity or crystallinity) than conventional syntheses.

In this review, we have summarized the examples published in the last three years on the preparation of COFs using microwaves as an energy source. These results update the review previously published on the same topic and give a complete perspective on the subject and its evolution over time.

The first aspect that draws attention is the limited number of works published in recent years of MAS of COFs compared with the total number of publications on the subject. The synthesis of some of the COFs requires long durations that are reduced to minutes with microwave use. In general, the materials obtained using microwaves have structures, crystallinities, and surfaces similar or superior to those obtained by conventional synthesis. Despite the obvious advantages, this type of technology is still a minority. The absence of this technology is not exclusive to COFs, but it appears in chemical synthesis, both organic and inorganic. Although certainty in this regard would require additional studies, we believe that the omission of microwave use could be due to the low proliferation of professional microwave equipment in synthesis laboratories. Only this type of equipment allows the achievement of controlled and reproducible synthesis conditions. In fact, we see a clear evolution in most of the materials contained in this article that have been prepared with this type of reactor. The microwave oven is a key aspect of this class of synthesis. Using specially designed equipment for chemical synthesis is very important for reproducible synthesis procedures over time and between laboratories. 

Although they have improved, a more detailed description of the synthesis procedures is still pending. In microwave synthesis, it is quite common that the published procedures do not include all the necessary information to be able to carry them out. Apart from temperature and power, we can cite as important examples the characteristics of the device, the volumes of the reactors, the volume of dissolution, whether there is stirring or not, how the cooling of the reactor is carried out, and other details. All these aspects are crucial in the synthesis of the materials.

In the materials that have been prepared, an evolution is clear. There is a reduction in the types of bonds, and, thus, in the type of COFs. The boronate esters and the materials with less stability have lost relevance, and no example has been found. By contrast, those based on imines or triazines, more stable compounds, maintain a prominent presence. Along with them, compounds with amide, C-C, or dioxin bonds offer interesting alternatives that will continue to have an outstanding contribution in the coming years. The 3D-COFs are still testimonial in line with their presence among COFs.

A third relevant aspect is a shift in the focus of the articles. The number of new materials, at least in the framework structure, has been reduced, but functionalization and the development of applications have gained importance. Thus, several studies use materials previously reported, such as a microwave that is modified or used in novel applications. Even in novel materials, the application becomes the main motivation of the articles. The applications include classics such as oil/water, gas separations or capture, and electrochemistry, including electrodes for water splitting or batteries. In addition the previously applied fields, COFs have also been applied in the biomedical field in the elimination of pollutants in dialysis processes, the release of genes, and the elimination of tumors. Another trend is the incorporation into membranes or the exploration of delaminated materials.

Some aspects of the microwave-assisted synthesis of COFs have not yet been addressed, and whose approach to the process would most benefit the discipline has not been determined. It is necessary to advance the understanding of the interaction of electromagnetic radiation with the reaction mixture. The choice of the solvent and its dielectric properties is of the greatest relevance. However, there is no information on these parameters in a generalized and accessible way for all compounds. The different hydrolysis and condensation processes necessary to obtain the crystalline structure of COFs will vary the properties of the reaction mixture so this behavior and its analysis and modelling require basic research and the interaction of multidisciplinary groups specialized in various areas as chemical, materials and physics.

Also, the advance in the use of microwave generators based on solid state technology, it would be convenient, recently it has arisen and offers interesting opportunities when developing low-cost, long lasting reactors that allow precise power control and that, due to their digital characteristics, can be programmed to obtain greater precision in the synthesis. We thought that the use of this technology is going to be the clue in the application of microwaves in laboratories and in consequence its extension in many other fields. In addition, we will start to see such benefits as these kinds of generators are included in commercial reactors and their possibilities are available to materials scientists

At last, in industrial scale-up and application processes may also be relevant a higher speed synthesis and lower energy consumption, both characteristics are bottlenecks when these advances in nanomaterials are transferred from the laboratory to a real plant application. In all cases, it is essential to have reproducible syntheses with the lowest possible energy and reagent costs. Microwaves can reduce reaction times, allowing an important reduction in the time in which the entire system needs to be kept at a high temperature and therefore in the total energy cost.

In conclusion, microwaves are a very interesting tool for preparing COFs. In recent years we have advanced towards a consolidation of the discipline. We hope that this article will be useful for all those groups that work in the field of COF synthesis to illustrate and identify new synthesis pathways.

## Figures and Tables

**Table 1:** COFs are summarized in the review with their main synthesis parameters and applications.

COF/POF	Knot/Vertice	Linker/Edge	MW Oven ^1^	Power [W]	t	P	T [°C]	Solvent ^2^	Pore Size [nm]	S_BET_ [m^2^ g^−1^]	Application	Ref.
LZU-1	TFB	NBPDA	n.a.	n.a.	30 min	n.a.	120	Et/ TFA	1.3	1388	n.a.	[43,44]
Au@LZU1	TFB	NBPDA	1500 MW high-pressure	n.a.	20 min	n.a.	120	Et/ TFMS	1.3	1066	n.a.	[43]
LZU-PEI	TFB	NBPDA	n.a.	n.a.	30 min	n.a.	120	Et/ TFMS/ PEI	n.a.	n.a.	gene vector	[44]
LZU-Cys	TFB	NBPDA	n.a.	n.a.	30 min	n.a.	120	Et/ TFMS Cys	n.a.	n.a.	gene vector	[44]
nCOP	TAPP	BPDA	CEM-D	n.a.	20 min	n.a.	120	DMF/ Me	n.a.	n.a.	pH-responsive cross-linkers	[45]
TAPB-TDA	TAPB	TDA	JC-101W	200	1 h	n.a.	n.a.	Dx/ Ms/ HAc	0.4	170	oil/water separation	[46]
TAPB-TFA	TAPB	TFA	JC-101W	200	1 h	n.a.	n.a.	Ms/ HAc	0.1	96	oil/water separation	[46]
TAB-DFP	TAPB	DFP	n.a.	n.a.	2 h	n.a.	100	Dx/ HAc	1.6	491	n.a.	[47]
TAB-MPPD	TAPB	MPPD	n.a.	n.a.	2 h	n.a.	100	Dx/ HAc	1.3	44	oil/water separation	[47]
TAPB-BPTA	TAPB	BPTA	JC-101W	200	1 h	n.a.	n.a.	Ms/ HAc	1.3	1653	interfacial bridging	[48]
DPCOF	DBPT	HKT + DADTA	Mon-Pro/ Mon-300	n.a.	3 h	n.a.	200	HCl/ MNp	n.a.	n.a.	Gas separation	[49]
IrNCOF	DBPT	HKT + DADTA	Mon-Pro/ Mon-300	n.a.	12 min + 2 h	n.a.	185 + 200	HCl/ MNp/ IrCl_3_	n.a.	n.a.	Gas separation	[49]
PtNCOF	DBPT	HKT + DADTA	Mon-Pro/ Mon-300	n.a.	3.5 h	n.a.	200	HCl/ MNp/ PtCl_2_	n.a.	n.a.	Gas separation	[49]
NiCOF	DBPT	HKT + DADTA	Mon-Pro/ Mon-300	n.a.	3 h	n.a.	200	HCl/ MNp/ NiCl_2_	n.a.	n.a.	Gas separation	[49]
TAPB-BTCA	TAPB	BTCA	ETHOS 1	200	5 h	n.a.	80	H_2_O/ HAc	n.a.	566	n.a.	[50]
CTF-0	TCB		CEM-D CEM-S	300	30 min	300 psi	110	TFMS	n.a.	0.5–5	photocatalysis	[51]
CTF-0	TCB		NN-GF33KB	800	20 min	n.a.	n.a.	CF_3_SO_3_H	0.67	282	n.a.	[52]
CTF-DCB	DCB		NN-GF33KB	800	20 min	n.a.	n.a.	CF_3_SO_3_H	1.1	672	n.a.	[52]
CTF-BPDCN	BPDCN		NN-GF33KB	220	3 h	n.a.	n.a.	CF_3_SO_3_H	2.0	536	n.a.	[52]
Pd@CTF	DCP		CEM-D	100	20 min	n.a.	250	Pd(acac)_2_	n.a.	904–1353	Oxygen reduction reaction	[53]
IrO_x_@CTF	DCP		CEM-D	100	3 × 10 min	n.a.	250	Ir_4_(CO)_12_	n.a.	918–1229	Hydrogen evolution reaction	[53]
PI-COF	TAPA	PMDA	AP-Mul	n.a.	2 h	n.a.	200	MPy	10–20	n.a.	n.a.	[54]
MA-PMDA	MA	PMDA	AP-Mul	n.a.	2 h	n.a.	200	MPy	10–20	n.a.	n.a.	[54]
MA-NTDA	MA	NTDA	AP-Mul	n.a.	2 h	n.a.	200	MPy	10–20	n.a.	n.a.	[54]
TH-COF	HHTP	TFPC	MDS-6 G	100	30 min	n.a.	70	Dx/ Ms/ TEA	2.2	1254	analysis of trace perfluorinated alkyl substances in water	[55]
COF-Co	HHTP	TFPN	Nova-2s	n.a.	72 h	n.a.	120	Co(CH_3_CO_2_)_2_	n.a.	n.a.	Potassium ion batteries	[56]
ACA-COF	ACA	BDB	n.a.	n.a.	3 h	n.a.	90	Et/ H_2_O	1.2 and 7.5	58	Removal of pollutants from dialysis wastewater	[57]
2D-COF	TAPB	BDB	n.a.	n.a.	2 h	n.a.	100	Et/ H_2_O	n.a.	30	CO_2_ capture	[58]

^1^ CEM-S: CEM S-Class Explorer; CEM-D: CEM Discover; AP-Mul: Anton Paar Multiwave Pro; Mon-Pro: monowave Pro; Mon-300: Monowave 300. ^2^ Dx: 1,4-dioxane, Ms: mesitylene, TEA: triethylamine, MPy: N-methyl-2-pyrrolidone, DMSO: dimethyl sulfoxide, Et: ethanol, HAc: acetic acid, TFA: trifluoroacetic acid, PEI: polyethyleneimine, Cys: cystamine, TFMS: trifluoromethanesulfonic acid, EtCly: ethylene glycol, MNp: 1-methylnaphtalene, DMF: dimethylformamide, Me: methanol. n.a: no available

## Data Availability

Not applicable.
